# Spatiotemporal Distribution of GABA_A_ Receptor Subunits Within Layer II of Mouse Medial Entorhinal Cortex: Implications for Grid Cell Excitability

**DOI:** 10.3389/fnana.2018.00046

**Published:** 2018-06-04

**Authors:** Nina Berggaard, Mohsen Seifi, Johannes J. L. van der Want, Jerome D. Swinny

**Affiliations:** ^1^Department of Clinical and Molecular Medicine, Faculty of Medicine and Health Sciences, Norwegian University of Science and Technology, Trondheim, Norway; ^2^Institute for Biomedical and Biomolecular Sciences, School of Pharmacy and Biomedical Sciences, University of Portsmouth, Portsmouth, United Kingdom

**Keywords:** grid cells, development, reelin, calbindin, parvalbumin, plasticity, interneuron

## Abstract

GABAergic parvalbumin-expressing (PV+) interneurons provide powerful inhibitory modulation of grid cells in layer II of the medial entorhinal cortex (MEC LII). However, the molecular machinery through which PV+ cells regulate grid cell activity is poorly defined. PV+ interneurons impart inhibitory modulation primarily via GABA-A receptors (GABA_A_Rs). GABA_A_Rs are pentameric ion channels assembled from a repertoire of 19 subunits. Multiple subunit combinations result in a variety of receptor subtypes mediating functionally diverse postsynaptic inhibitory currents. Whilst the broad expression patterns of GABA_A_R subunits within the EC have been reported, those expressed by individual MEC LII cell types, in particular grid cells candidates, stellate and pyramidal cells, are less well described. Stellate and pyramidal cells are distinguished by their selective expression of reelin (RE+) and calbindin (CB+) respectively. Thus, the overall aim of this study was to provide a high resolution analysis of the major (α and γ) GABA_A_R subunits expressed in proximity to somato-dendritic PV+ boutons, on RE+ and CB+ cells, using immunohistochemistry, confocal microscopy and quantitative RT-PCR (qPCR). Clusters immunoreactive for the α1 and γ2 subunits decorated the somatic membranes of both RE+ and CB+ cells and were predominantly located in apposition to clusters immunoreactive for PV and vesicular GABA transporter (VGAT), suggesting expression in GABAergic synapses innervated by PV interneurons. Although intense α2 subunit-immunopositive clusters were evident in hippocampal fields located in close proximity to the EC, no specific signal was detected in MEC LII RE+ and CB+ profiles. Immunoreactivity for the α3 subunit was detected in all RE+ somata. In contrast, only a sub-population of CB+ cells was α3 immunopositive. These included CB-α3 cells which were both PV+ and PV−. Furthermore, α3 subunit mRNA and immunofluorescence decreased significantly between P 15 and P 25, a period implicated in the functional maturation of grid cells. Finally, α5 subunit immunoreactivity was detectable only on CB+ cells, not on RE+ cells. The present data demonstrates that physiologically distinct GABA_A_R subtypes are selectively expressed by CB+ and RE+ cells. This suggests that PV+ interneurons could utilize distinct postsynaptic signaling mechanisms to regulate the excitability of these different, candidate grid cell sub-populations.

## Introduction

The internal representation of space is generated from a complex interplay between functionally distinct cell types within the hippocampal formation. Grid cells within layer II of the medial entorhinal cortex (MEC LII) are an important component of this spatial system. During exploration, each grid cell fires at multiple discrete locations that make up a triangular array covering the entire space available (Hafting et al., 2005). While the exact mechanisms underlying the striking activity pattern are yet to be understood, recent evidence suggests that grid cells include both stellate cells and pyramidal cells, which are the two main principal cell types in MEC LII (Domnisoru et al., 2013; Tang et al., 2014; Sun et al., 2015). These cell types can be chemically distinguished by their respective expression of reelin (RE+) and calbindin (CB+; Fuchs et al., 2015; Donato et al., 2017; Witter et al., 2017). Furthermore, studies have shown that principal cells within this layer to a large degree communicate via GABAergic interneurons (Couey et al., 2013; Fuchs et al., 2015), and that in particular parvalbumin-expressing (PV+) interneurons play a crucial role in maintaining the periodic firing pattern in grid cells (Buetfering et al., 2014; Miao et al., 2017).

Different classes of PV+ interneurons entrain the activity of principal cells by releasing GABA selectively onto specific sub-domains of principal cells, such as somato-proximal dendritic or axon-initial segment compartments (Klausberger et al., 2003). In turn, principal neurons target an array of GABA receptors to their sub-cellular compartments in order to effectively transmit such GABA-mediated information (Farrant and Nusser, 2005; Fritschy and Panzanelli, 2014). Since PV+ interneurons are characterized by their fast spiking activity (Klausberger et al., 2003), rapid processing of GABA-mediated synaptic transmission is integral to PV-mediated regulation of excitability. Within the CNS, the rapid effects of GABA are predominantly mediated by GABA_A_ receptors (GABA_A_Rs; Xiang et al., 1998).

GABA_A_Rs are integral-membrane complexes, composed of five different proteins, or subunits, which assemble to form an anion-permeable ion channel (Farrant and Nusser, 2005). Although only five subunits are required to form a functional receptor, 19 different variants are currently known, namely α1–6, β1–3, γ1–3, δ, ε, θ, π and ρ1–3 (Farrant and Nusser, 2005). The variety of potential subunit combinations gives rise to a number of receptor subtypes, which diverge according to the anatomical expression, as well as physiological and pharmacological properties (Farrant and Nusser, 2005). Collectively, GABA_A_R subtype diversity significantly magnifies the repertoire of GABA-mediated inhibition within a particular brain region. In the MEC, the broad GABA_A_R subunit expression spectrum has been previously described (Drexel et al., 2013; Hörtnagl et al., 2013). However, the layer and cell type specific expression profiles of individual GABA_A_R subunits remain to be elucidated, especially in terms of the principal cell types. Such data are crucial given the established role that GABA-mediated inhibition plays in regulating neuronal excitability.

The functional maturation of grid cell activity is distinct from other navigational and memory encoding cell types, such as head direction cells, place cells and boundary responsive cells (Langston et al., 2010; Tan et al., 2017). In the rodent, the fraction of cells exhibiting stable grid pattern activity has previously been reported to increase significantly between postnatal days (P) 19–22 (Wills et al., 2010, 2012). Given the putative role of GABAergic-mediated transmission via PV+ interneurons in this process, and the likely importance of GABA_A_R-mediated signaling for PV+ interneurons, it is reasonable to speculate that GABA_A_R subtype plasticity may also occur during this time window. Therefore, the overall aim of this study was to provide a high resolution analysis of the major (α and γ) GABA_A_R subunits expressed in proximity to somato-dendritic PV boutons, on RE+ and CB+ principal cells of MEC LII, and determine whether such expression changes significantly during a period implicated in grid cell maturation.

## Materials and Methods

All procedures involving animal experiments were approved by the Animal Welfare and Ethical Review Body of the University of Portsmouth and were performed by a personal license holder, under a Home Office-issued project license, in accordance with the Animals (Scientific Procedures) Act, 1986 (UK) and associated procedures.

### Tissue Preparation for Immunohistochemistry

Adult male C57BL/6J mice, aged P 60, were used to determine the native GABA_A_R subunit expression patterns. For quantitative analyses, mice aged P 15 (*N* = 6) and P 22 (*N* = 6) were used. The tissue was perfusion-fixed as follows: anesthesia was induced with isoflurane and maintained with pentobarbitone (1.25 mg/kg of bodyweight; i.p.). The animals were perfused transcardially with 0.9% saline solution for 2 min, followed by 12 min fixation with a fixative consisting of 1% paraformaldehyde and 15% v/v saturated picric acid in 0.1 M phosphate buffer (PB), pH 7.4. After the perfusion, the brains were carefully dissected from the skull and post fixed over night at room temperature in the same perfusion fixative. The following day, the brains were rinsed in 0.1 M PB, after which 50 μm-thick sagittal sections were prepared using a vibratome (Leica VT 1000). The sections were thoroughly washed in 0.1 M PB to remove any residual fixative and then stored in a solution containing 0.1 M PB and 0.05% sodium azide until further processing.

### Immunohistochemistry

**Figure 1:**
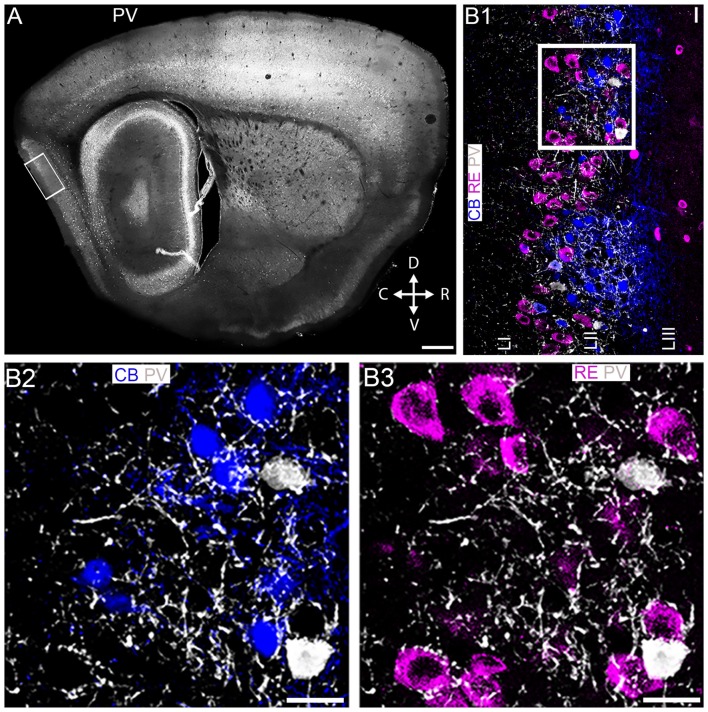
Association of parvalbumin, RE+ and CB+ neurons in layer II of the medial entorhinal cortex (MEC LII). **(A)** Overview of parvalbumin (PV) immunoreactivity, at the mediolateral and dorsoventral position in a sagittal section of the mouse brain. The rectangle represents the main area of focus for this study. **(B1)** Magnified view of the boxed area in **(A)** showing an overlay image of immunoreactivity for PV (white), CB (blue) and RE (magenta). **(B2,B3)** Magnified view of the boxed area in **(B1)** demonstrating strong innervation of PV+ puncta around most CB+ pyramidal **(B2)** and RE+ stellate **(B3)** cells. D, dorsal; V, ventral; C, caudal; R, rostral. Scale bars: **(A)** 200 μm; **(B1–B3)**10 μm.

Tissue sections containing an elongated hippocampus (see Figure 1A) corresponding to 2.5–3.5 mm from the midline were used for all reactions. For immunolabeling of the GABA_A_R α2 and γ2 subunits, a proteolytic antigen retrieval method (Watanabe et al., 1998; Lorincz and Nusser, 2008) was employed as follows: tissue sections were warmed to 37°C for 10 min in 0.1 M PB and subsequently incubated in a solution containing 1 mg/ml pepsin (Sigma, UK), in 0.2 M HCl for a further 10 min. All sections were then washed in 50 mM TRIS-buffered saline (TBS) containing 0.03% Triton X-100 (TBS-TX) for 30 min. Non-specific binding of the secondary antibodies was minimized by incubating the sections in TBS-TX containing 20% normal horse serum (S-2000, Vector Laboratories Inc., Burlingame, CA, USA) for 2 h. Sections were incubated in a cocktail of primary antibodies over night at 4°C (Table 1). The next day, the sections were washed with TBS-TX for 30 min after which they were incubated at room temperature in a cocktail of an appropriate mixture of secondary antibodies, conjugated with DyLight TM 405, Alexa Fluor 488, indocarbocyanine (Cy3) and indodicarbocyanine (Cy5), all provided by Jackson Immunoresearch, for 2 h. The sections were washed in TBS-TX for 30 min after which they were mounted on glass slides, air dried and coverslipped using Vectashield mounting medium (H-1000, Vector Laboratories Inc., Burlingame, CA, USA).

**Table 1:** Overview of primary antibodies used in the study.

Antibody	Host	Dilution	Source	Specificity/Reference
Parvalbumin	Chicken	1:2000	Synaptic Systems Cat# 195 006, RRID:AB_2619887	Labeling pattern as published with other antibodies
VGAT	Goat	1:3000	Frontier Institute (VGAT-Go-Af620)	Miyazaki et al. (2003)
Reelin	Mouse	1:1000	Millipore Cat# MAB5364, RRID:AB_2179313	Labeling pattern as published with other antibodies
Calbindin	Mouse	1:1000	Swant Cat# 300, RRID:AB_10000347	Celio et al. (1990)
MAP2	Chicken	1:250	Aves Labs Cat# MAP, RRID:AB_2313549	Labeling pattern as published with other antibodies
GABA_A_R α1	Rabbit	1:3000	Synaptic Systems Cat# 224 203, RRID:AB_2232180	Wisłowska-Stanek et al. (2013) and Seifi et al. (2014)
GABA_A_R α2	Rabbit	1:500	Werner Sieghart, antigen sequence α2L amino acids 322–357. R # 28/16 Bleed # 01/10/2002	Pirker et al. (2000) and Seifi et al. (2014)
GABA_A_R α3	Rabbit	1:5000	Synaptic Systems Cat# 224 303, RRID:AB_2619931	Fritschy and Mohler (1995) and Seifi et al. (2014)
GABA_A_R α4	Rabbit	1:1000	Werner Sieghart antigen sequence, α4N amino acids 1–9. Rabbit #21/7, bleed #04/10/1999	Maguire et al. (2014)
GABA_A_R α5	Rabbit	1:5000	Synaptic Systems Cat# 224 503, RRID:AB_2619944	Pirker et al. (2000) and Seifi et al. (2014)
GABA_A_R γ2	Rabbit	1:3000	Synaptic Systems	Fish et al. (2013)

### Antibody Specificity

The specificity of all the GABA_A_R subunit antibodies has been verified previously using GABA_A_R subunit gene-deleted mice (Seifi et al., 2014). Method specificity was also tested by omitting the primary antibodies in the incubation sequence. To confirm the absence of cross reactivity between IgGs in double and triple immunolabeling experiments, some sections were processed through the same immunohistochemical sequence, except that only an individual primary antibody was applied with the full complement of secondary antibodies.

### Image Acquisition

Sections were examined with a confocal laser-scanning microscope (LSM710; Zeiss, Oberkochen, Germany) using a Plan Apochromatic 63× DIC oil objective (NA 1.4, pixel size 0.13 μm). Z-stacks were used for routine evaluation of the labeling. All images presented represent a single optical section. These images were acquired using sequential acquisition of the different channels to avoid cross-talk between fluorophores, with the pinholes adjusted to one airy unit. Images were processed with the software Zen 2009 Light Edition (Zeiss, Oberkochen, Germany) and exported into Adobe Photoshop. Only brightness and contrast were adjusted for the whole frame, and no part of a frame was enhanced or modified in any way.

### Semi-Quantitative Analysis of the Changes in MEC LII GABA_A_R Subunit Expression Between P 15 and P 22

One of the aims of the study was to investigate any association between changes in GABA_A_R subunit expression levels during a period implicated in grid cells maturation, which evidence indicates occurs during the third and fourth postnatal weeks. We therefore quantified the changes in GABA_A_R subunit expression in the MEC, between two time-points, P 15 and P 22, at both the protein and mRNA levels, using immunofluorescence intensity and quantitative RT-PCR (qPCR; detailed below), respectively. Differences in the fluorescence intensity of MEC LII GABA_A_R subunit immunoreactivity was quantified in tissue from P 15 and P 22 mice, using previously published protocols (Gunn et al., 2013). The imaging and quantification was performed as follows: within a tissue section, a field of view (FOV) was selected within the dorsal portion of MEC LII containing the strongest GABA_A_R subunit signal. The dimensions of each FOV were 134.8 μm × 134.8 μm × 1 μm in the X-Y-Z planes. Within a FOV, the fluorescence intensity of the GABA_A_R subunit was measured using ImageJ software. This was repeated in 1–2 tissue sections per animal, and 4–6 animals per age, per subunit. An average value was then computed for all FOVs. This average value for an individual animal was then considered an N of 1.

### GABA_A_R Subunit mRNA Expression Analysis Using Quantitative RT-PCR (qPCR)

Male mice aged P15 and P25 were killed by cervical dislocation before their brains were rapidly removed. To ensure all of MEC was included, sagittal sections corresponding to regions 2 and 4 mm from the midline were dissected using a brain matrix and snap frozen in liquid nitrogen and stored at −80°C until further use. The frozen tissue was then homogenized in appropriate amounts of lysis buffer from which RNA was extracted using an RNeasy mini kit (Qiagen, 74104) according to the manufacturer’s protocol. The quality and quantity of the extracted RNA in each tissue was examined with spectrophotometry (Thermo Scientific™ NanoDrop™). The reverse transcription was performed according to our previously published protocols (Seifi et al., 2014). Equal amounts of RNA from each tissue was reverse-transcribed into first strand cDNA in the following reaction: 2 μl of 10× M-MuLV Reverse Transcriptase Reaction Buffer which included, in the final concentration, 50 mM Tris–HCl, 10 mM DTT, 75 mM KCl and 3 mM MgCl_2_ (BioLabs), 0.1 mM Oligo(dT)18 Primer (Thermo Fisher Scientific), 1 mM dNTP Mix (Thermo Fisher Scientific), 0.5 μl of M-MuLV reverse transcriptase (Applied Biosystems), and 0.5 μl of riboLock (Thermo Fisher Scientific). Each reaction was then incubated at 37°C for 2 h. After running the PCR, the values were exported into Excel and the final concentrations were calculated. Quantitative PCR (qPCR) amplification was performed in 96-well plates in a master mix for probes (Roche, Burgess Hill, United Kingdom) and run on a LightCycler R 96 System (Roche). The qPCR amplifications for the mouse Gabra1 (assay ID: Mm00439046_m1), Gabra3 (assay ID: Mm01294271_m1), Gabra4 (assay ID: Mm00802631_m1), Gabra5 (assay ID: Mm00621092_m1), Gabrg2 (assay ID: Mm00433489_m1) and Pvalb (assay ID: Mm00443100_m1) genes were performed using pre-designed TaqMan primers/probes purchased from Life Technologies (Thermo Fisher scientific). Gapdh (assay ID: Mm99999915_g1) gene expression was used as the housekeeping gene in every reaction. The qPCR cycling conditions entailed 95°C for 10 min and 40 cycles of 95°C for 15 s and 60°C for 60 s (LightCycler R 96 System, Roche). Standard curves were generated for each gene using serial dilutions of a known amount of mRNA extracted from each organ which were then reverse transcribed into cDNA. Each measurement was performed in duplicate and each Ct value was then converted into ng mRNA using linear regression analysis of the standard curve (Microsoft Excel). Each ng mRNA value was then normalized against the ng housekeeping gene level within the same sample and the mean mRNA levels for every sample was finally calculated and compared across all experimental groups.

### Statistical Analysis

All quantitative data are presented as the mean ± SEM. Statistical differences between means were assessed using an unpaired Student’s *t*-tests, using GraphPad Prism software. A *P*-value less than 0.05 was considered statistically significant.

## Results

The overall aim of the study was to: (1) determine the spatiotemporal expression patterns of specific GABA_A_R subunits on MEC LII RE+ stellate and CB+ pyramidal cells, in relation to PV immunoreactive boutons, in the adult mouse; and (2) investigate possible subunit plasticity during a period implicated in grid cell pattern maturation, namely P 15 to P 25.

### CB+ and RE+ MEC LII Cells Receive Dense PV+ Input

At the outset, we assessed the association of PV immunoreactivity (Figure 1A) with CB and RE immunoreactivity within MEC LII. While RE+ cells presented as a continuous band along the superficial part of the LII dorsoventral axis, CB+ cells were located in prominent clusters, in particular in the dorsal portion, spanning the entire depth of LII. Although PV+ interneurons were greatly outnumbered by principal cells, PV immunoreactivity was prominent throughout LII and appeared strongly associated with CB+ and RE+ profiles (Figure 1B1). Indeed, high resolution inspection revealed that both CB+ (Figure 1B2) and RE+ (Figure 1B3) somata and dendrites were intensely decorated with PV+ varicosities.

### The GABA_A_R α1 Subunit Is Expressed by Both RE+ and CB+ MEC LII Cells

**Figure 2:**
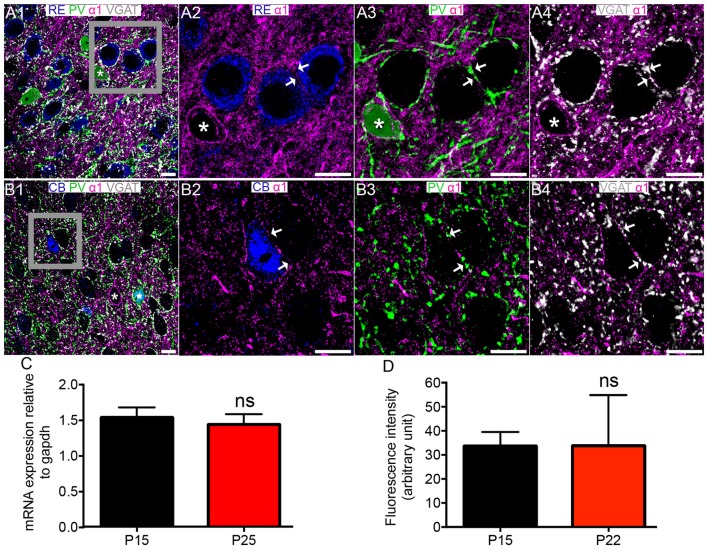
Association of the GABA_A_R α1 subunit with RE+ and CB+ neurons in MEC LII. **(A1)** shows an overview of immunoreactivity for RE (blue), PV (green), α1 (magenta) and VGAT (white). **(A2–A4)** Magnified view of the boxed region in **(A1)** showing immunoreactivity for α1 surrounding RE+ cells, apposed to PV and VGAT immunoreactivity (arrows). Note strong α1 immunoreactivity bordering the PV+ cell soma (asterisk). **(B1)** shows an overlay of immunoreactivity for CB (blue) with α1, PV and VGAT as in the **(A1)**-panel. Note strong α1 immunoreactivity surrounding the immunonegative cell body (asterisk) and the CB and PV double immunopositive cell (star). **(B2–B4)** Magnified view of the boxed region in **(B1)** showing immunoreactivity for a CB+ cell soma, bordered by α1 immunoreactivity, which is in part apposed to PV and VGAT immunoreactivity (arrows). **(C)** Relative amounts of α1 mRNA within the entire MEC at P 15 and P 25. **(D)** Average fluorescence intensity of α1 immunolabeling at P 15 and P 22. The bars represent means ± SEM. ns, *P* > 0.05, unpaired Student’s *T* test; **(C)** *N* = 7 animals, **(D)** *N* = 6 animals. Scale bars: **(A,B)** 10 μm.

In line with previous reports depicting an overview of GABA_A_R subunit expression within the EC (Wisden et al., 1992; Pirker et al., 2000; Hörtnagl et al., 2013), intense immunoreactivity for the α1 subunit was widely distributed within this brain region (Figures 2A1,B1). Close inspection confirmed that within MEC LII, membrane-bound α1 subunit immunoreactivity encircled somata immunopositive for RE (Figure 2A2) and PV (Figure 2A3). However, α1 subunit immunoreactivity on the PV+ GABAergic interneurons was noticeably more intense compared to the signal evident on RE+, and other putative principal cells. This gradient of expression, across principal cells and interneurons, is in keeping with other cortical regions (Gao and Fritschy, 1994). Somatic α1 subunit immunoreactive clusters were often closely apposed to clusters immunopositive for the vesicular GABA transporter (VGAT), a predictor of GABAergic synapses (Figure 2A4). CB+ somata also exhibited clustered α1 subunit immunoreactivity on their perisomatic surfaces (Figure 2B2). Qualitatively, in contrast to RE+ profiles, there was comparatively less association between α1 subunit clusters and PV (Figure 2B3) and VGAT immunoreactive varicosities (Figure 2B4) on CB+ somata.

There were no significant differences in the levels of MEC α1 subunit mRNA (*P* = 0.6429, unpaired Student’s *T* test; *N* = 7 animals; Figure 2C) between the ages of P 15 and P 25 and immunoreactivity (*p* = 0.9825, unpaired Student’s *T* test; *N* = 6 animals; Figure 2D) between P 15 and P 22.

### The Somatodendritic Surfaces of CB+ and RE+ MEC LII Cells Are Devoid of GABA_A_R α2 Subunit Expression

**Figure 3:**
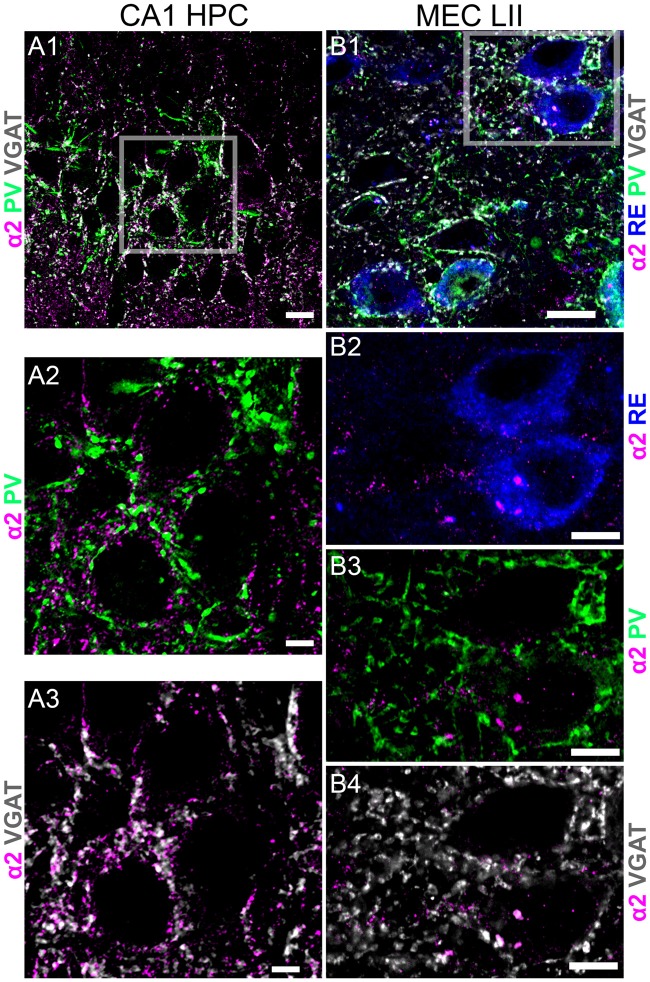
Comparison of immunoreactivity of the GABA_A_R α2 subunit between *stratum pyramidale* of CA1 and MEC LII. **(A1)** An overlay of immunoreactivity for α2 subunit (magenta), PV (green) and VGAT (white) within *stratum pyramidale* of CA1. **(A2)** Magnified view of the boxed area in **(A1)** showing puncta immunoreactivity for PV (green) contacting clusters immunoreactive for the α2 subunit (magenta). **(A3)** Magnified view of the boxed area in **(A1)** showing puncta immunoreactivity for VGAT (white) contacting clusters immunoreactive for the α2 subunit (magenta). **(B1)** An overlay of immunoreactivity for α2 subunit (magenta), PV (green) and VGAT (white) and RE within MEC LII. **(B2)** Magnified view of the boxed area in **(B1)** showing somatic immunoreactivity for RE (blue) and clusters immunoreactive for the α2 subunit (magenta). Note the sparsity of α2 subunit immunoreactive clusters within this region, compared to the adjacent CA1 sub-field. Furthermore, there is no α2 subunit immunoreactivity associated with the somatodendritic domains of RE profiles. **(B3,B4)** Magnified view of the boxed area in **(B1)** showing immunoreactivity for α2 subunit with PV (green) and VGAT (white), respectively. Scale bars: **(A1,B1)** 10 μm; **(A2,A3,B2–B4)** 5 μm.

A previous study reported only moderate GABA_A_R α2 subunit expression in the mouse EC (Hörtnagl et al., 2013). We therefore used another brain region known to be enriched with the α2 subunit, namely *stratum pyramidale* of the CA1 region of the hippocampus, as a positive control to verify our experimental conditions. In agreement with published reports (Pirker et al., 2000; Hörtnagl et al., 2013), α2 subunit immunoreactivity within this region was closely associated with boutons immunopositive for PV and VGAT, suggesting expression in GABAergic synapses innervated by PV terminals (Figures 3A1–A3). However, under identical experimental conditions, no specific α2 subunit signal was associated with the somatodendritic surfaces of RE+ cells (Figures 3B1–B4) or CB+ cells (data not shown). This suggests that α2-GABA_A_Rs may have an insignificant role in GABA-mediated regulation of the excitability of these candidate grid cells.

### The GABA_A_R α3 Subunit Immunoreactivity Is Preferentially Associated RE+ Rather Than CB+ Cells in the MEC LII

**Figure 4:**
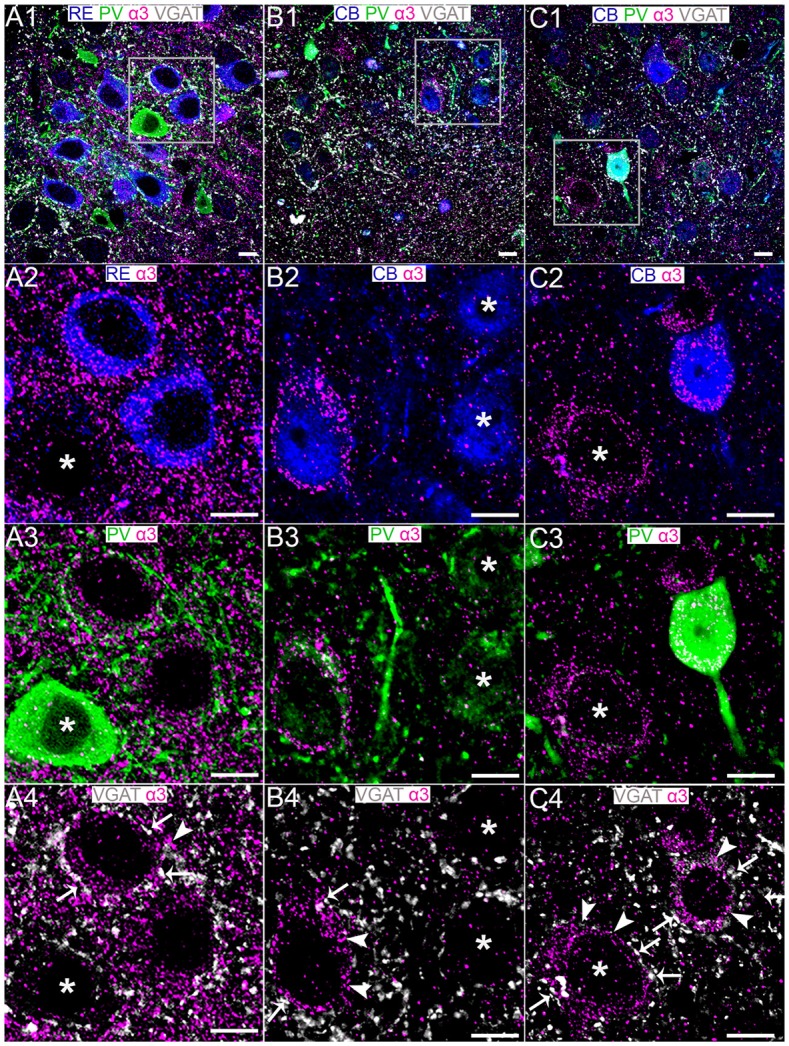
Association of the GABA_A_R α3 subunit with RE+ and CB+ neurons in MEC LII. **(A1)** shows an overview of immunoreactivity for RE (blue), PV (green), α3 (magenta) and VGAT (white). **(A2–A4)** Magnified view of the boxed region in **(A1)** showing α3 subunit immunoreactive clusters on RE+ cell somata, some of which are apposed to PV and VGAT immunoreactivity. Note limited α3 immunoreactivity associated with neighboring PV+ soma (asterisk). **(B1)** shows an overlay of immunoreactivity for CB (blue) with α3, PV and VGAT as in the **(A1)**-panel. **(B2–B4)** Magnified view of the boxed region in **(B1)** showing the association of the α3 subunit with a subset of CB+ cell somata. Some of this α3 immunoreactivity was associated with PV and VGAT immunolabeling. The asterisks in **(B2)** indicate CB+ cells devoid of α3 subunit immunoreactivity. Note that the CB+/α3 soma in **(B2)** is immunonegative for PV. **(C1)** shows an overlay of immunoreactivity for CB (blue), α3, PV and VGAT as in the **(B1)**-panel. **(C2–C4)** Magnified view of the boxed region in **(C1)** showing the association of the α3 subunit with a CB+ cell soma that is PV immunopositive. Note α3 immunoreactivity associated with CB immunonegative, putative RE+ cell (asterisks). In **(A4,B4,C4)** the arrows indicate instances of close VGAT-α3 subunit association, suggestive of a synaptic locus of expression for this subunit. The arrowheads indicate instances of disparate VGAT-α3 subunit association, suggestive of an extrasynaptic locus of expression for this subunit. Scale bars, 10 μm.

Dense α3 subunit immunoreactivity was evident in the region of the MEC LII containing RE+ cells (Figure 4A1). High resolution inspection revealed that α3 subunit immunoreactive clusters ensheathed RE+ somata (Figure 4A2). These α3 subunit immunopositive clusters on RE+ somata were also closely apposed to PV+ (Figure 4A3) and VGAT+ varicosities (Figure 4A4). In contrast to the consistent association of α3 subunit immunoreactivity with most, if not all RE+ cells, only isolated CB+ cells showed somatic immunoreactivity for this subunit (Figures 4B1,B2). Since the somata of these CB+/α3 immunopositive cells were qualitatively large and devoid of PV immunoreactivity (Figure 4B3), this population of CB+/α3 cells most likely represent principal cells rather than interneurons. Interestingly, other similar CB+ putative principal cells did not display α3 immunoreactivity (Figure 4B2; see asterisks). The CB+/α3 subunit immunoreactive cells were contacted by puncta immunopositive for PV (Figure 4B3) and VGAT (Figure 4B4). A further population of CB+/α3 subunit immunoreactive cells were themselves immunopositive for somatic PV (Figures 4C1–C4). Based on the neurochemical phenotype, this population of CB-PV-α3 cells most likely represent interneurons.

**Figure 5:**
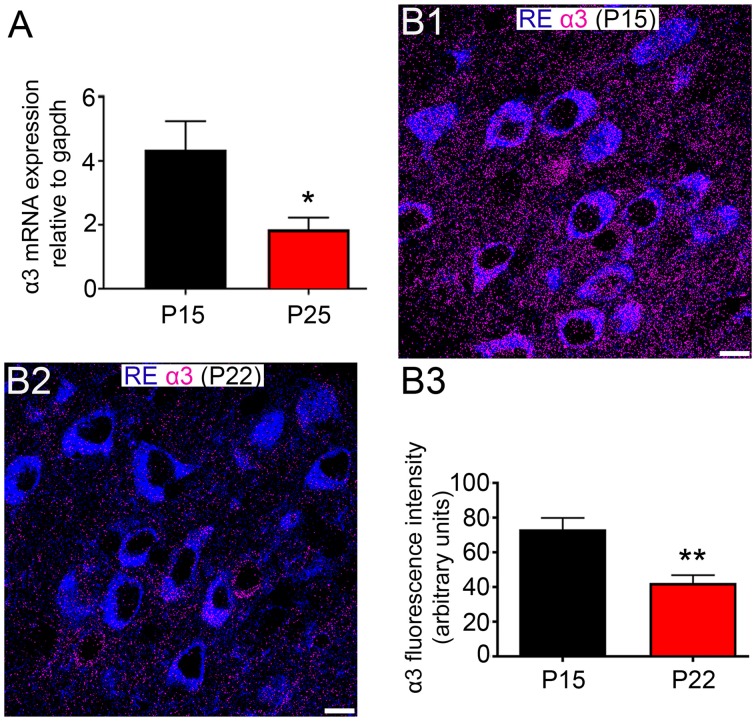
Changes in α3 subunit expression in the MEC of animals between the ages of P 15 and P 25. **(A)** Quantification of the relative amounts of α3 mRNA within the entire MEC at P15 and P 25, normalized to the housekeeping gene *gapdh*. **(B1,B2)** Representative images of the comparative intensity of α3 subunit immunoreactivity (magenta) associated with RE+ cells in dorsal MEC LII of tissue sections from animal aged P 15 and P 22, respectively. Note the significant decrease in the intensity of α3 subunit signal between the ages. Note also that samples were processed and imaged under identical conditions. **(B3)** Quantification of the fluorescence intensity of α3 immunoreactivity associated with RE+ cells in MEC LII of tissue sections from animal aged P 15 and P 22. The bars represent means ± SEM. *P < 0.05, unpaired Student’s *T* test; *N* = 7 animals, **p < 0.01, unpaired Student’s *T* test; *N* = 5 animals. Scale bars: **(B1,B2)** 10 μm.

Remarkably, the α3 subunit exhibited significant expression plasticity during the postnatal window of P 15 to P 25. Indeed, α3 mRNA levels in MEC showed a significant decrease (57.2%) between P 15 and P 25 (*P* = 0.0241, unpaired Student’s *T* test; *N* = 7; Figure 5A). A significant decrease in expression (43%) between P 15 and P 22 was evident at the protein level as well (*P* = 0.008, unpaired Student’s *T* test; *N* = 5; Figures 5B1–B3).

### The GABA_A_R α5 Subunit Immunoreactivity Is Preferentially Associated With CB+ Rather Than RE+ Cells in the MEC LII

**Figure 6:**
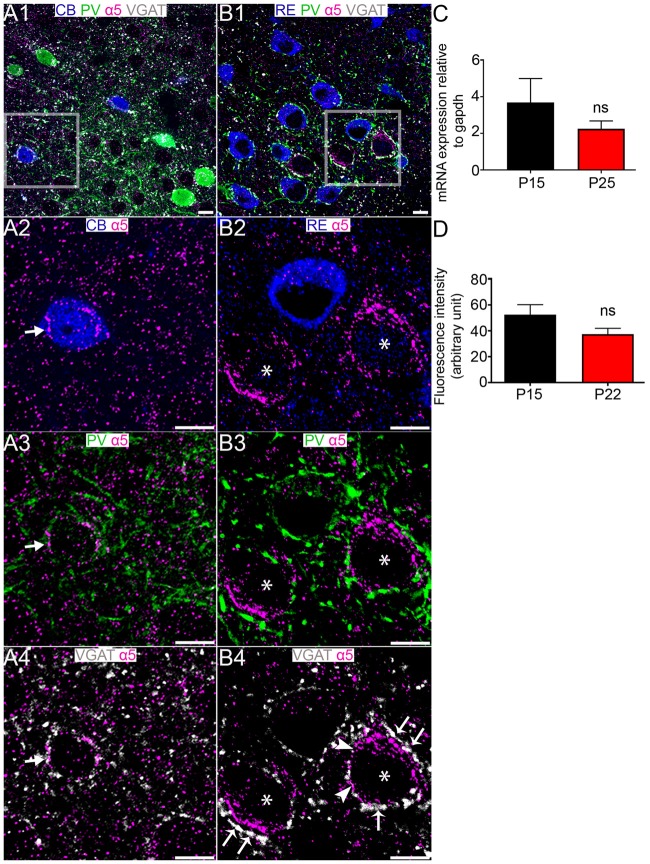
Association of the GABA_A_R α5 subunit with CB+ and RE+ neurons in MEC LII. **(A1)** shows an overview of immunoreactivity for CB (blue), PV (green), α5 (magenta) and VGAT (white). **(A2–A4)** Magnified view of the boxed region in **(A1)** showing α5 subunit immunoreactive clusters on a CB+ cell soma, in close proximity to PV and VGAT immunoreactivity (arrows). **(B1)** shows an overlay of immunoreactivity for RE (blue) with α5, PV and VGAT as in the **(A1)**-panel. **(B2–B4)** Magnified view of the boxed region in **(B1)** showing a lack of association of the α5 subunit with the RE+ cell soma. Note strong immunoreactivity for α5 on the putative CB+ somata (asterisks). In **(B4)** the arrows indicate instances of close VGAT-α5 subunit association, suggestive of a synaptic locus of expression for this subunit. The arrowheads indicate instances of disparate VGAT-α5 subunit association, suggestive of an extrasynaptic locus of expression for this subunit. **(C)** Relative amounts of α5 mRNA within the entire MEC at P 15 and P 25. **(D)** Mean fluorescence intensity of α5 immunolabeling at P 15 and P 22. The bars represent means ± SEM. ns, *P* > 0.05, unpaired Student’s *T* test; **(C)** *N* = 7 animals, **(D)** *N* = 6 animals. Scale bars: **(A,B)** 10 μm.

No specific signal for the α4 subunit was detectable in MEC LII, or in association with RE+ and CB+ cells (data not shown). Immunoreactivity for the α5 subunit decorated the perisomatic surfaces of CB+ cells (Figures 6A1–A4). However, we could not detect any specific α5 subunit immunoreactivity associated with RE+ cells, despite intense α5 subunit in the close vicinity of RE+ profiles which are likely to be CB+ cells (Figures 6B1–B4). There were no significant differences in the levels of α5 subunit expression between P 15 and P 25 at the mRNA level (*P* = 0.3179, unpaired Student’s *T* test; *n* = 7, Figure 6C) or between P 15 and P 22 at the fluorescence intensity level (*P* = 0.1224, unpaired Student’s *T* test; *n* = 6; Figure 6D).

### The GABA_A_R γ2 Subunit Is Differentially Expressed on the Somato-Dendritic Compartments of RE+ and CB+ MEC LII Cells

**Figure 7:**
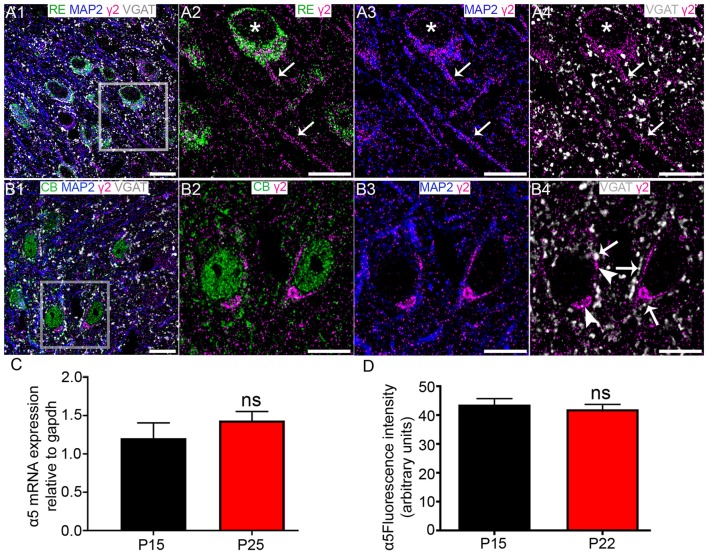
Association of the GABA_A_R γ2 subunit with CB+ and RE+ neurons in MEC LII. **(A1)** shows an overview of immunoreactivity for RE (green), MAP2 (blue), γ2 (magenta) and VGAT (white). **(A2–A4)** Magnified view of the boxed region in **(A1)** showing γ2 subunit immunoreactive clusters on a RE+ cell soma (asterisks). Note immunoreactivity for γ2 on nearby putative dendrites (arrows). **(B1)** shows an overlay of immunoreactivity for CB (green) with γ2, MAP2 and VGAT as in the **(A1)**-panel. **(B2–B4)** Magnified view of the boxed region in **(B1)** showing clusters of γ2 immunoreactivity on CB+ cell somata and dendrites. In **(B4)** the arrows indicate instances of close VGAT-γ2 subunit association, suggestive of a synaptic locus of expression for this subunit. The arrowheads indicate instances of disparate VGAT-γ2 subunit association, suggestive of an extrasynaptic locus of expression for this subunit. **(C)** Relative amounts of γ2 mRNA within the entire MEC at P 15 and P 25. **(D)** Average fluorescence intensity of γ2 immunolabeling at P 15 and P 22. The bars represent means ± SEM. ns, *P* > 0.05, unpaired Student’s *T* test; **(C)** *N* = 7 animals, **(D)** *N* = 6 and 4 animals for P 15 and P 22, respectively. Scale bars: **(A,B)** 10 μm.

Robust γ2 subunit immunoreactivity was associated with both RE+ cells (Figure 7A1) and CB+ cells (Figure 7B1). However, the signal appeared to be targeted to different sub-cellular domains of these cell types. Immunoreactivity for the γ2 subunit appeared to be evenly distributed across RE+ somata (Figure 7A2) and dendrites, identified by immunoreactivity for the dendritic marker protein microtubule associated protein 2 (MAP2; Figure 7A3). This γ2 subunit immunoreactivity was closely associated with puncta immunoreactive for VGAT (Figure 7A4). In contrast, γ2 immunoreactivity appeared to be comparatively more associated with the somatic than putative dendritic surfaces of CB+ cells, appearing as bright clusters within CB+ somata (Figures 7B2,B3). These CB+/γ2 subunit clusters were also strongly associated with puncta immunopositive for VGAT (Figure 7B4). There were no significant changes in the levels of γ2 mRNA (*p* = 0.3399, unpaired Student’s *T* test; *N* = 7, Figure 7C) and mean fluorescence intensity (*p* = 0.8013, unpaired Student’s *T* test; *N* = 6 and 4 for P 15 and P 22, respectively; Figure 7D).

## Discussion

The current study provides a high resolution description of the identity and location of individual GABA_A_R subunits within the MEC LII GABAergic circuitry composed of PV+, RE+ and CB+ cells. The data indicate that some subunits (α1; γ2) are widely expressed on RE+ and CB+ somatic surfaces in close proximity to PV and VGAT immunopositive puncta. In contrast, other subunits (α3; α5) are preferentially expressed by these principal cell types. Collectively, this suggests that PV+ interneurons could employ different GABA_A_R subtypes to entrain the activity of RE+ and CB+ cells, thereby diversifying the PV-mediated modulatory patterns of these candidate grid cells.

### PV+ Basket Cells Innervate RE+ and CB+ Cells in MEC LII

Previous studies have indicated that grid cells include both stellate and pyramidal cells. Yet, there is a discrepancy between physiological and immunohistochemical data whether PV+ cells inhibit pyramidal cells in MEC LII (Fuchs et al., 2015; Armstrong et al., 2016; Ray et al., 2017; Witter et al., 2017). Congruent with previous immunohistochemical studies (Armstrong et al., 2016; Ray et al., 2017), we found that the dorsal part of MEC LII contains CB+ cells arranged in patches (islands) with complementary RE+ (ocean) cells, both of which are heavily innervated by PV+ basket terminals. We cannot rule out an absence of PV+ innervation of a subset of CB+ neurons, and, since not all pyramidal cells are CB+ (Fuchs et al., 2015), its pyramidal counterpart. However, our results suggest that the majority of CB+ cells are inhibited by PV+ cells. Such PV-dependent inputs onto RE+/CB+ somatodendritic compartments likely reside alongside those originating from other classes of interneurons, for example, cholecystokinin-expressing basket cells. Therefore, we cannot rule out that the GABA_A_R subunit expression patterns discussed below also apply to such inputs.

### Fast Phasic GABAergic Inhibition of Cells in MEC LII Are Most Likely Mediated Via α1-GABA_A_Rs and Not α2-GABA_A_Rs

GABA_A_R-mediated postsynaptic inhibitory currents (IPSCs) display a range of kinetic properties thereby magnifying the versatility of GABA-mediated signaling throughout the nervous system. Indeed, the temporal profiles of GABA_A_R-IPSCs range from rapid transient currents (fast phasic), slower transient currents (slow phasic), to persistently active currents (tonic; Farrant and Nusser, 2005). Convergent lines of evidence indicate that the identity of the subunits composing the receptor subtypes is a reasonable predictor of the IPSC kinetics. GABA_A_Rs composed of α1/2 subunits invariably exhibit fast phasic IPSCs whilst α3-GABA_A_Rs generally mediate slower phasic inhibition (Eyre et al., 2012). Given that the signature property of PV+ interneurons is their rapid modulation of target cells, they most likely rely predominantly on an appropriately rapid postsynaptic signaling mechanism to ensure fidelity of information transfer. As such, α1/2-GABA_A_Rs are generally widely expressed by cortical principal neurons which are modulated by PV+ interneurons (Kasugai et al., 2010; Kerti-Szigeti and Nusser, 2016). In agreement with a previous report of widespread α1 immunoreactivity in the mouse EC (Hörtnagl et al., 2013), we observed dense α1 labeling within MEC LII. The α1 immunoreactive clusters were dispersed throughout the neuropil containing RE+ and CB+ cells, as well as being located on the somata of these principal cell types, often in apposition to PV and VGAT immunoreactive clusters which are generally representative of synaptic GABAergic release sites. Collectively, this provides compelling evidence that α1-GABA_A_Rs contributes to PV-mediated IPSCs in both RE and CB principal cells.

Given the general widespread expression of the GABA_A_R α2 subunit in principal cells throughout diverse brain regions (Nyíri et al., 2001; Prenosil et al., 2006; Kerti-Szigeti and Nusser, 2016), it is remarkable that both principal cell types of MEC LII are devoid of immunoreactivity for this subunit, at least on their perisomatic membranes. Since the antibodies for RE and CB do not label the entire dendritic arbors of these cell types, it is not feasible to unequivocally rule out expression of this subunit on all the domains of these cells. Furthermore, since the GABA_A_R α2 subunit expression has been shown to be developmentally down-regulated (Peden et al., 2008), we cannot rule out expression in these cell types during early postnatal periods. Nevertheless, in terms of perisomatic inhibitory regulation, the data suggest that α1-GABA_A_Rs are the principal mediators of PV-dependent fast phasic IPSCs on these cell types.

Despite the association of GABA_A_R α1 subunit expression with MEC LII principal cells, it was noticeable that relatively higher levels of signal were located on PV+ interneurons within this region (Figure 2). These most likely represent PV+, CB+/PV+ and CB-/PV- putative interneurons. In contrast to the presentation of the signal on the principal cells as individually identifiable clusters, the labeling pattern on these interneurons appeared as signal that was continuous on somato-dendritic plasma membranes. This enhanced density of GABA_A_R α1 subunit expression on interneurons, compared to principal cells, is reflective of such enrichment in these cell types in other cortical regions (Gao and Fritschy, 1994). Thus, despite the differences in the GABA_A_R subunit expression profiles for this region, compared to adjacent cortical divisions discussed above, those for interneurons appear to be consistent with other domains. This is important since interconnected interneurons, especially (PV+) basket cells, are important for network synchrony and generating theta and gamma network oscillations (Cobb et al., 1995; Baude et al., 2007). The robust expression of α1-GABA_A_Rs within PV+ cellular networks suggests a prominent role for such signaling molecules in synchronizing the LII network in a similar manner.

### Distinct GABA_A_R Subtypes Could Facilitate Cell-Type Specific Control of Grid Cells by PV+ Basket Cells

As discussed above, the subunit combinations composing the GABA_A_R determines the properties of the IPSCs they generate. Thus, an individual PV interneuron whose axon terminals innervate synapses containing different GABA_A_R subtypes will likely impart contrasting forms of inhibition across such diverse synapse populations. The current data suggest that different GABA_A_R subtypes could be targeted to those RE+ and CB+ perisomatic domains innervated by PV+ interneurons. Indeed, all observed RE+ somata were enriched with immunoreactivity for the GABA_A_R α3 subunit (Figure 3A). In contrast, we found α3 immunoreactivity in only a subset of CB+ cells devoid of PV immunoreactivity, and in CB+ cells co-expressing PV (Figure 3B). CB is expressed by both principal cells and interneurons in MEC LII (Fujimaru and Kosaka, 1996; Kitamura et al., 2014; Fuchs et al., 2015), and it is likely that the population of CB-PV-α3 cells observed are interneurons. Not all PV+ interneurons exhibited α3 subunit signal, however. We are unable to distinguish, neurochemically, whether the two groups of CB+/PV immunonegative cells either associated with, or devoid of α3 immunoreactivity do in fact represent distinct populations of pyramidal principal cells. However, a previous study has reported that RE+ is co-expressed with wolframin1 (another marker for CB+ cells, see, Kitamura et al., 2014) in a subset of intermediate stellate and intermediate pyramidal cells, and to a limited extent in pyramidal cells (Fuchs et al., 2015). Considering we found α3 immunoreactivity associated with virtually all RE+ cells, it is possible that these CB+/α3 cells are also RE+ and belong to distinct populations of LII principal cells. Taken together, whilst it is clear that the GABA_A_R α3 subunit is associated with most, if not all RE+ cells, there is the possibility that some CB+ principal cells do not utilize α3-GABA_A_Rs to mediate PV-dependent somatic inhibition. This is important because the GABA_A_R α3 subunit has been shown to impart complex patterns of inhibition, in the form of both phasic and tonic inhibitory currents (Farrant and Nusser, 2005). However, in contrast α1/2-GABA_A_Rs that mediate fast phasic IPSCs, α3-GABA_A_Rs mediate slower phasic IPSCs due to slow channel activation and deactivation rates (Barberis et al., 2007; Eyre et al., 2012). Thus, perisomatic PV-dependent IPSCs on RE+ cells are most likely mediated by α1/3-GABA_A_Rs. This is likely to manifest in heterogeneous phasic currents. In contrast, IPSCs on a sub-population of CB+ principal cells may be generated by receptors devoid of the α3 subunit, thus resulting in PV-mediated regulation that is distinct from RE+ cells.

**Figure 8:**
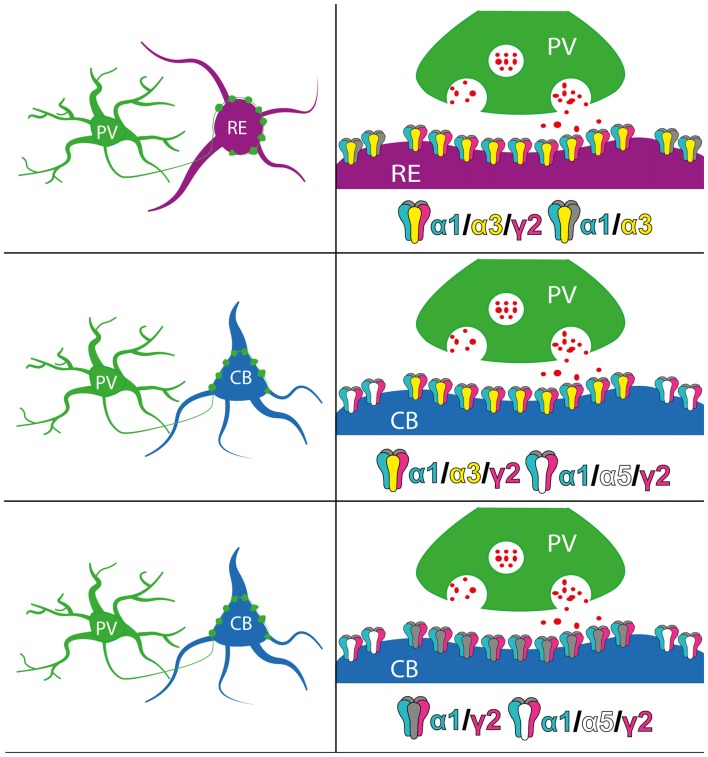
Schematic summary of the perisomatic expression of GABA_A_R subunits on RE+ and CB+ neurons in MEC LII. Red dots indicate GABA molecules.

Furthermore, α3-GABA_A_Rs have been shown to underlie the sustained tonic inhibitory currents in addition to phasic events (Marowsky et al., 2012). Generally, tonic inhibitory currents are generated by receptors located on extrasynaptic domains (Glykys and Mody, 2007). Apart from the GABA_A_R α3 subunit (and α4 which we could not detect), the α5 subunit is the other major α subunit implicated in generating tonic inhibitory currents (Prenosil et al., 2006). It was noticeable that clusters immunoreactive for the GABA_A_R α1 subunit were not associated with puncta immunopositive for the synaptic marker protein VGAT, suggesting a proportion of extrasynaptic α1-GABA_A_Rs in both RE+ and CB+ cells. Since the α5 subunit, but not the α3 subunit, was consistently expressed throughout all CB+ cells, it is reasonable to speculate that α1–5-GABA_A_Rs underlie the majority of tonic inhibitory currents in these cells. In contrast, given the consistent expression of the α3 subunit in RE+ but not CB+ cells, the data predict that α1–3-GABA_A_Rs underlie the majority of tonic inhibitory currents in RE+ cells. If so, this translates to both phasic and tonic forms of GABA_A_R-mediated inhibition being distinct on these candidate grid cell populations. As such, individual GABAergic interneurons underlying this inhibitory regulation of these cell types, such as PV basket cells, are likely to induce varying postsynaptic signaling pathways thereby imparting cell type-specific regulation of their overall excitability, summarized in Figure 8.

### GABA_A_R Subunit Specific Plasticity During the Period Associated With Grid Cell Activity Stabilization

The qPCR analysis revealed a significant increase in PV mRNA expression in MEC between P 15 and P 25. However, changes at the protein level assessed by quantifying the intensity of PV immunoreactivity selectively in dorsal MEC LII, were not statistically significant. A likely explanation is that the tissue processed for qPCR analysis was not restricted to MEC LII. Thus, this increase in mRNA levels is most likely due to the delayed development of PV in deeper layers of MEC compared to LII (Donato et al., 2017).

Out of all the GABA_A_R assessed for changes in expression during the chosen postnatal window, only the α3 subunit showed significant changes, at both the mRNA and protein levels. Given the disparate expression pattern of this subunit across RE+ and CB+ cells, and its unique contribution to GABA-mediated inhibition, the decrease in its expression during development is likely to differentially impact on the maturation of perisomatic IPSCs on these cell types. Since the expression of the α1 subunit did not change during this period, the increased ratio of α1:α3 subunits could result in the preferential acceleration, during grid cell activity stabilization, of the time constants for IPSCs generated on RE+ cells. It is debatable whether this directly contributes to grid cell activity specifically, or adhering more to a general maturational trend observed also in other brain regions.

In conclusion, the data provides a high resolution depiction of the identity of the major GABA_A_R subunits expressed by the two principal cell types of the MEC LII and the sub-cellular location of these subunits in proximity to one of their major local inputs to these cells, PV+ interneurons. This expression analysis indicates that PV-mediated regulation of MEC LII RE+ and CB+ could be via distinct subsets of GABA_A_Rs. Given the importance of these cell types as candidates for grid cells, the data sheds new light on the molecular machinery which could contribute to one of the most salient contributions of MEC LII to overall brain function, namely spatial navigation.

## Data Availability

The raw data supporting the conclusions of this manuscript will be made available by the authors, without undue reservation, to any qualified researcher.

## Author Contributions

NB, MS and JS designed research and performed research. NB, MS, JW and JS analyzed research and wrote the article.

## Conflict of Interest Statement

The authors declare that the research was conducted in the absence of any commercial or financial relationships that could be construed as a potential conflict of interest.
